# Comments on the published meta-analysis of antibiotic resistance in hospital-acquired ESKAPE-E infections in low- and lower-middle-income countries

**DOI:** 10.1080/22221751.2022.2073829

**Published:** 2022-06-10

**Authors:** Zahra Hadi, Forough Goodarzi, Amine Dalir, Mohammad Sholeh

**Affiliations:** a Department of microbiology, school of basic sciences, Karaj Branch, Islamic Azad university, Karaj, Iran; b Department of Bacteriology, Faculty of Medical Sciences, Tarbiat Modares University, Tehran, Iran; c Department of Microbiology, Faculty of Medicine, Iran University of Medical Sciences, Tehran, Iran; d Department of Bacteriology, Pasteur Institute of Iran, Tehran, Iran

To the editor

While studying Antibiotic resistance, we considered the article entitled “Antibiotic resistance in hospital-acquired ESKAPE-E infections in low- and lower-middle-income countries: a systematic review and meta-analysis” by O. Ayobamia [1]. According to the article, the search was performed in the electronic databases EMBASE, Web of Science, and Global Index Medicus for studies published between January 2010 and September 2020 in English, French, German, Spanish, Portuguese languages. After screening and selection, data extraction of 163 included articles was performed. The extracted data were included the primary and secondary outcomes and the following study characteristics first author, year of publication, study period, country, city, WHO region, continent, national income level category, study design, regional or national representativeness, hospital setting, age group, hospital type, antimicrobial susceptibility testing (AST) guideline used, and HAI type. In exclusion criteria, it is stated that articles with less than 10 isolates sample size have been excluded from the meta-analysis. This limitation is suggested to use when there is a significant small study effect (Egger test [2] or Begg test [3]), in which case deleting these articles with a smaller sample size could be helpful to reduce the small study effect bias.

The quality assessment of the included articles has been done, but these results have not been used in the analyzes. The quality assessment result could be used in a subgroup or meta-regression analysis to investigate the heterogeneity source.

Due to the article’s title, the primary outcome of this meta-analysis is the proportion of ESKAPE + E isolates exhibiting marker-antibiotic resistance among all clinical ESKAPE + E isolates. To answer this research question, defining antibiotic resistance is critical. All or most included articles in this meta-analysis should be cross-sectional articles, and they should have reported the guideline that was used to interpret AST results. In fact, antibiotic resistance is a relative definition that is defined according to the agreed breakpoints to interpret AST results. And due to the existence of multiple guidelines and significant differences in critical points for the interpretation of MIC in them, the definition of resistance can be very different. For example, the breakpoint for *S. aureus* AST interpretation in the EUCAST guideline, which is more commonly used in Europe, MIC > 2, is used as the breakpoint for vancomycin. In contrast, the CLSI guideline uses MIC ≥ 16 as the breakpoint [4,5]. This difference in the definition of resistance becomes more important when it includes antibiotic resistance in different countries, especially in different continents. Countries usually interpret antibiotic resistance results with a guideline, although this is not always the case. Therefore, to perform antibiotic resistance meta-analysis, it is necessary to perform subgroup analysis based on the guideline or, if possible, based on the breakpoints used to interpret the AST results. In a more comprehensive method, MIC data analysis methods can be used to perform a meta-analysis because, in this case, the definitions are mainly the same, and the results will be more reliable and accurate.

In this meta-analysis, there is no mention of publication bias, and no analysis is done to investigate the publication bias. The funnel plot is commonly used to check publication bias. However, this method is not quantitative, and its interpretation is inaccurate. Egger’s regression test and Begg’s rank test can be used to assess publication bias and the small-study effect.
